# A Cohort Study on Clinical Features and Microbiological Profile in Neonatal Sepsis in Southwest of Iran

**DOI:** 10.1002/hsr2.71398

**Published:** 2025-10-28

**Authors:** Fatemeh Javanmardi, Amir Emami, Hamidreza Parsa, Zahra Hashemi, Atila Erami, Zeynab Noeizad

**Affiliations:** ^1^ Biostatistics Department Shiraz Medical School, Shiraz University of Medical Sciences Shiraz Iran; ^2^ Burn and Wound Healing Research Center Shiraz University of Medical Sciences Shiraz Iran; ^3^ Department of Child and Infant Health Shiraz University of Medical Sciences Shiraz Iran; ^4^ Neonatal Research Center Shiraz University of Medical Sciences Shiraz Iran

**Keywords:** *Escherichia coli*, Neonates, Sepsis, *Staphylococcus aureus*

## Abstract

**Background and Aims:**

A bloodstream infection known as “sepsis” with “Neonatal sepsis,” which is a bloodstream infection associated with high morbidity and mortality. To function effectively and apply the appropriate treatments, it is necessary to comprehend the bacterial etiology, antibiotic resistance profile, and risk factors. The purpose of this study was to determine the variables that contribute to neonatal sepsis and the pattern of antibiotic resistance.

**Methods:**

This multicenter cohort study was conducted among neonates and their mothers admitted to two gynecological and maternity hospitals in Shiraz, Iran. Heel‐prick venous blood was obtained from all neonates after which it was cultured and the outcome used to group the study participants as positive and negative study groups. The antibiotic susceptibility profile of isolated bacteria against commonly prescribed antibiotics was then determined using standard protocol. An independent sample t‐test/chi‐square where appropriate was used to determine the association between the study variables.

**Results:**

Among a cohort of 592 newborns, 271 (45.77%) were admitted with early onset sepsis, meanwhile, 321 patients (54.22%) had negative culture results for sepsis detection. The maternal age (t = 5.55 *p*‐value < 0.05), gestational age (t = 4.98 *p*‐value < 0.05), and mode of delivery (F = 17.42 *p*‐value < 0.05) were maternal characteristics that associated with neonatal sepsis. Lethargy (F = 6.52 *p*‐value = 0.010), low nutrition (F = 14.21 *p*‐value < 0.05), vomit (F = 11.23 *p*‐value = 0.001), hypothermia (F = 7.21 *p*‐value = 0.04), and intubation (F = 15.62 *p*‐value < 0.05) were significantly more frequent in the positive group than in the negative group. The predominant bacterial species isolated were *Staphylococcus aureus (S. aureus)* (60.14%) and *Coagulase‐negative staphylococci* (CoNS) as compared to gram‐negative (*Escherichia coli and Klebsiella pneumonia)*. Ninety‐one percent of the *S. aureus* isolates were resistant to penicillin (83 isolates, 50.9%) and ampicillin (67 isolates, 41.1%).

**Conclusion:**

Both neonatal and maternal factors were associated with neonatal sepsis in these cohort of neonates admitted to health facilities in Iran, as such strengthening risk‐based prevention strategies and improving institutional delivery practices are crucial in reducing morbidity and mortality among such vulnerable group.

## 1 Introduction

Neonatal sepsis is a systemic infection occurring in infants during the first 28 days of life and it is an important cause of morbidity and mortality of newborns. Significant progress has been made in the past few years to reduce the mortality rate of children under the age of five and eliminate cause‐specific mortality in the vulnerable group [1]. However, neonatal sepsis account for two‐thirds of under‐5 mortalities [2]. Neonatal sepsis is classified based on the time of onset as early onset sepsis (EOS) occurring in the first 3 days of life or late onset sepsis (LOS) after the third day of life [3].

As an invasive infections account for 1.4 million infant deaths worldwide [4]. However, this percentage fluctuates according to diagnostic techniques and national context. A significant portion of interventions in the first days have focused on reducing these sepsis [5]. Neonatal sepsis is one of the main causes of death in low and middle‐income countries accounting for two‐thirds of under‐5 mortalities in these regions. These mortality rates have been reported to be10 to 36 times greater in low and middle‐income countries than in high income countries [6].

First birth, multiple pregnancies, mother's education, rural location, and male sex were found to be factors associated with a higher risk of death due to neonatal sepsis [7]. Even with this knowledge there is still a need for effective interventions since the reasons for deaths among this age group are varied [8]. Abnormal heart rate, temperature fluctuations, and respiratory distress are early indicators of high‐risk status in neonates [9]. In addition to clinical symptoms and laboratory results, blood cultures are the gold standard for diagnosing bloodstream infections. However, these diagnostic methods are not well‐established or consistently applied in healthcare services for infants in low‐income countries. Even within a certain locale, the etiology of sepsis can vary by region and change over time. These disparities are commonly associated with changes in societal practices and trends in antibiotic consumption [10].

Antibiotic usage during pregnancy may have direct effects on the unborn child or may influence the development of resistance by altering the neonatal microbiota. The increasing prevalence of drug‐resistant pathogens, complicates the management of neonatal sepsis by reducing the efficacy of standard antibiotics, limiting therapeutic options, and increasing the risk of persistent infections in vulnerable newborns [11].

Multidrug‐resistant (MDR) colonization and subsequent infections have become increasingly common among neonates admitted to Newborn Intensive Care Units (NICUs) over recent decades, driven by selective antibiotic pressure and environmental reservoirs in these high‐risk settings. While not all neonates with sepsis require NICU admission, these units are critical for managing severe cases, where MDR pathogens are more prevalent [12]. The danger of antibiotic‐resistant bacteria is increasing in the NICU, putting the most vulnerable newborns at higher risk of illness and death. Nowadays, antibiotic resistance is thought to be responsible for 30% of newborn sepsis mortality [13]. Although treating newborn sepsis with antibiotics is crucial, there are conflicting views on the selection of antimicrobial drugs in various clinical settings due to the absence of local data on antimicrobial resistance and unique bacterial profiles [14]. Hence, clinicians are constrained to utilize data from alternative contexts predominantly from more advanced nations to guide their therapeutic judgments [15]. This study therefore, aimed at determining the prevalence of early onset sepsis, bacterial agents responsible for neonatal sepsis and their antimicrobial resistance patterns to commonly prescribed antibiotics, and neonatal and maternal variables associated with sepsis among neonates hospitalized in Shiraz, Iran.

## 2 Methods

### 2.1 Study Design and Participants

This prospective multicenter cohort study was conducted among newborns and their mothers admitted to the Hafez and the Zeinabiyeh Hospitals in Shiraz, Iran during the January 2023‐December 2023. These tertiary health‐care are hospitals have well‐equipped NICUs and provides specialized maternity services to clientele within its catchment area. The study population comprised of neonates who were clinically diagnosed as having early onset sepsis based on the Integrated Management of Neonatal and Childhood Illness (IMNCI) criteria for identifying sepsis in neonates. Early‐onset sepsis (EOS) refers to a bloodstream infection occurring in a newborn within the first 72 h of life [16]. These neonates presented with at least one clinical symptom, such as fever (> 38°C), hypothermia (< 36°C), rapid breathing (> 60 breaths/min), severe chest indrawing, poor feeding, movement only when stimulated, convulsions, lethargy, or unconsciousness, and at least two hematological indicators, including an absolute neutrophil count (< 1500 or > 7500 cells/mm³), erythrocyte sedimentation rate (ESR) (> 15 mm/h), or platelet count (< 150 or > 440 × 10³/mm³). Following the observation of clinical symptoms, a blood culture was performed to confirm the presence of infection. The study stud population was categorized as positive if blood cultures showed presumptive growth and negative if there is no culture growth. All eligible neonates meeting these criteria were enrolled to determine the sample size for this group during the study period. Neonates who received antibiotics before blood culture collection and mothers who receive antibiotics before delivery were excluded.

### 2.2 Determination Sample Size and Data Collection

The sample size was determined by convenience, including all eligible neonates admitted during the study duration.

A self‐designed data collection tool was used to collected data on maternal sociodemographic, obstetrics and clinical variables and neonatal information, such as gestational age, multiple births (twins or more), delivery method, premature rupture of membranes (amniotic sac breaking before labor starts), recurrent abortions, history of urinary tract infections, and delivery types, including cesarean and spontaneous vaginal delivery [17]. Neonatal socio‐demographic and clinical signs like baby's birth weight, sex, hypothermia, head circumstance (measured as the maximum occipitofrontal circumference of the neonate's head, height (recumbent length) measured as the supine length of the neonate from the crown of the head to the heel, first and fifth minute apgar (the first minute score determines the infant's well‐being during labor and the 5‐min assessment informs the healthcare provider of the baby's well‐being after birth), vomiting, convulsion (epileptic fits occurring from birth to the end of the neonatal period), low nutrition (a neonate receives insufficient intake of milk) [18], skin disorder, congenital anomalies, tachycardia, apnea (a sudden cessation of breathing that lasts for at least 20 s or is accompanied by bradycardia or oxygen desaturation (cyanosis) in an infant younger than 37 weeks gestational age), tachypnea, jaundice (total serum bilirubin exceeding 12 mg/dL in term infants, or reaching levels of ≈ 20 mg/dL) [19], intubation and duration of admission [9, 20, 21, 22, 23]. All neonates were administered broad‐spectrum antibiotics as a first‐line treatment after the blood collection as part of the routine treatment of sepsis at the facilities and neonates were monitored until discharge or 3 death. Broad‐spectrum antibiotic treatment is replaced with targeted antibiotic once the causative organisms has been identified through the blood cultures [14].

### 2.3 Specimen Collection and Processing

A butterfly needle (ref) was used to obtain 1–3 ml of a heel‐prick venous blood aseptically from every study subject by the attending physician and transferred into two 20 ml brain heart infusion blood culture bottle and stored at a temperature of 35°C–37°C. All blood samples transferred to the Bacteriology of the Shiraz University of Medical Sciences, Iran within 2 h for culturing and antibiotic sensitivity test.

### 2.4 Bacteriological Culture and Identification of Bacterial Species

All blood culture bottles were incubated under aerobic conditions in an automated blood culture system at a temperature between 35°C and 37°C for 7 days until growth or otherwise. Positive growth cultures indicated by microbial growth via signals such as turbidity or gas production were removed for further processing. A sterile syringe was used aseptically to withdrawn sample from each positive bottle and inoculated onto Blood agar (Oxoid Ltd, Basingstoke, Hampshire, UK), MacConkey agar (Oxoid Ltd, Basingstoke, Hampshire, UK), and Chocolate agar (Oxoid Ltd, Basingstoke, Hampshire, UK) plates in accordance with the standard operating procedures (SOPs) at the facility. Blood agar and MacConkey agar plates were incubated aerobically at 35°C–37°C for 24 h, while Chocolate agar plates were incubated in a microaerophilic environment (5%–10% CO_2_) for 48–72 h. Visible growth was assessed by signs such as hemolysis, colony formation, turbidity, or gas bubbles. Positive blood cultures showing growth were aseptically subcultured onto Blood agar, MacConkey agar, and Chocolate agar using the quadrant streak method to isolate pure colonies. Gram staining was then performed on isolated colonies to determine Gram reaction and cellular morphology as an initial step in pathogen characterization. The quadrant streak method was employed to obtain pure colonies of the bacterial isolates. In brief, a sterile loop was used to obtain isolated pure colonies of the inoculum, which were streaked in a pattern that dilutes the bacterial concentration across the agar surface, allowing individual colonies to grow in isolation. Plates were incubated as described: Blood agar and MacConkey agar plates aerobically at 35°C–37°C for 24 h, and Chocolate agar plates in a microaerophilic environment (5%–10% CO_2_) for 48–72 h. Visible growth was assessed, and well‐isolated colonies were selected for further characterization to ensure purity. This method follows standard microbiological protocols. After obtaining pure colonies and performing Gram staining, biochemical tests were conducted to identify key pathogens. For gram‐positive cocci, such as *Staphylococcus aureus* and CoNS, the identification tests included catalase (to differentiate staphylococci from streptococci), coagulase (to distinguish *S. aureus* from CoNS), and mannitol fermentation (for further confirmation of *S. aureus*). For Gram‐negative bacilli, such as *Escherichia coli* and *Klebsiella pneumoniae*, tests included oxidase, indole production, citrate utilization, urease, and triple sugar iron (TSI) agar reactions, following standard protocols as described by Leber (2020) [19]. When biochemical tests were inconclusive or required confirmation (e.g., for atypical isolates or to resolve ambiguous results), automated identification systems were employed. These systems provided rapid and standardized identification based on biochemical profiles. The automated identification system used in this study was the VITEK 2 Compact system (bioMérieux, France). An automated identification system using the VITEK 2 Compact system (bioMérieux, France) was employed to identify bacterial organisms for those in which the biochemical tests were inconclusive or required confirmation (e.g., for atypical isolates or to resolve ambiguous results). The VITEK 2 system provides a rapid and standardized identification of bacterial organism by analyzing biochemical reactions and metabolic profiles using a standardized card‐based system, ensuring high accuracy and reproducibility. This system is widely used in clinical microbiology laboratories and aligns with standard protocols. Negative cultures indicated by no culture growth after 5 days were discarded [24, 25].

### 2.5 Antimicrobial Susceptibility Testing

The antibiotic susceptibility of isolated pure culture colonies was performed using a modified Kirby‐Bauer disk diffusion method, following the guidelines established by the Clinical and Laboratory Standards Institute (CLSI) [26].

A sterile wire forceps was used to harvest, 3–5 well‐isolated colonies. The suspended colony was then adjusted to achieve an inoculum turbidity equivalent to 0.5 McFarland standard and swabbed onto a Mueller Hinton Agar (MHA) plate. Antibiotic disks infused with commonly prescribed antibiotics used in treating neonatal sepsis at the facility were carefully positioned on the MHA plate and incubated at a temperature of 35°C to 37°C for a period of 16 to 18 h. The concentrations of antibiotics utilized per disk (Oxoid UK) in this investigation included amoxicillin (Clindamycin (30 mg), Cefoxitin (30 mg), Erythromycin (5 mg), Oxacillin (30 mg), Penicillin (30 mg), Trimethoprim‐sulfamethoxazole (5 mg), Tetracycline (5 mg), Ampicillin (30 mg), Ceftriaxone (30 mg), Chloramphenicol (30 mg), Gentamicin (30 mg), Ciprofloxacin (30 mg), Amoxicillin‐clavulanate (30 mg), Tetracycline (30 mg), Amikacin (5 mg), Ceftazidamide (30 mg), Chloramphenicol (5 mg). The zones of inhibition were measured with a caliper and categorized as sensitive, intermediate, or resistant as recommended by the National Committee for Clinical Laboratory Standards (NCCLS) guidelines [24, 27].

### 2.6 Statistical Analysis

All completed data collection tools were entered into and analyzed with the Statistical Package for the Social Sciences v 22 (IBM, USA). For descriptive statistics, the frequencies and percentages were reported for categorical variables, whiles the mean and standard deviation were reported for continuous variables. Independent sample t‐test and Chi‐square test appropriate were performed to determine the association between groups. A significance level of 0.05 was deemed as statistically significant for every test conducted.

### 2.7 Ethical Consideration

A written consent to participate was obtained from the mothers/guardians of the neonate. The Shiraz University of Medical Sciences Review Board approved this study as minimal‐risk research, based on data collected during routine clinical practice (Ethics Code: R.SUMS.REC.1400.830).

## 3 Results

### 3.1 Maternal Sociodemographic, Clinical and Obstetric Characteristics

The mean age of the mothers in the positive group was 30.70  ±  6.37 years and 31.15  ±  17.73 years in the negative group. In the positive group, the majority of mothers (68.83%, 186) resided in urban areas, whereas in the negative group, 82.24% (*n* = 264) were from urban areas. A statistically significant difference was observed in the mean body weight between the two groups (t = 5.55 *p*‐value < 0.05). The gestational age was significantly higher in the positive group than in the negative group (t = 4.98 *p* < 0.05). The majority, 69.74% (189) of the neonates in the positive group and 84.11% (270) in the negative group were delivered through a caesarian section. There was a statistically significant difference between type of delivery and neonatal sepsis (F = 17.42 *p*‐value < 0.05). 57.94% (186) mothers in the negative group received perinatal care services at least once during their pregnancy, compared to 229 mothers (84.50%) in the positive group. Antibiotic prophylaxis was used by 31.36% (85) of mothers group positive cases as compared with 1.86% (6) in the group negative. Additionally, mothers of children who were impacted by a blood infection had a greater frequency of prior abortions and urinary tract infections in comparison to mothers of children who were not affected by sepsis. A history of multiple births was seen in 23.05% (74) of mothers without neonatal sepsis, and 17.71% (48) of mothers with neonatal sepsis. This difference was not statistically significant (*p*‐value = 0.18). Chorioamnionitis (F = 5.19 *p*‐value = 0.02), prolonged delivery (F = 14.10 *p*‐value ≤ 0.05), and tachycardia (F = 0.37 *p*‐value ≤ 0.05) were statistically significant and clinically relevant factors associated with a higher frequency of neonatal sepsis in the positive blood culture group compared to the negative group. All other variables were not statistically significant (Table 1).

**Table 1:** Maternal characteristics during pregnancy and childbirth.

Variables	Group positive (*n* = 271)	Group negative (*n* = 321)	*p* value	Statistics
Mother's age (years)	30.70 ± 6.37	31.15 ± 17.73	< 0.05	t = 0.38
Gestational age (weeks)	35.19 ± 5.33	33.08 ± 3.83	< 0.05	T = 0.55
Residence of the mother (Urban: Rural)	186: 85 (68.63%: 31.36%)	264: 57 (82.24%: 17.75%)	0.02	F = 4.89
Mode of delivery (Cesarean: Vaginal birth)	189: 82 (69.74%: 30.25%)	270: 51 (84.11%: 15.88%)	< 0.05	F = 17.42
Type of pregnancy (Twin birth and above)	48 (17.71%)	74 (23.05%)	0.18	F = 4.79
Received perinatal care (1–3 visits: more than 3 visits)	229: 42 (84.50%: 15.49%)	186: 135 (57.94%: 42.05%)	0.32	F = 26.42
Using prophylaxis antibiotic	85 (31.36%)	6 (1.86%)	< 0.05	F = 100.97
Smoking	3 (1.10%)	0 (0.00%)	0.05	
Diabetic Mellitus	25 (9.22%)	44 (13.70%)	0.09	F = 2.86
Positive vaginal culture for GBS	3 (1.10%)	0 (0.00%)	0.05	F = 1.18
History of UTI	6 (2.21%)	0 (0.00%)	< 0.05	F = 1.18
Premature rupture of the amniotic sac	29 (10.70%)	22 (6.85%)	0.13	F = 3.95
Multi‐abortion	32 (11.80%)	23 (7.16%)	0.05	F = 3.75
Invasive procedures	2 (0.73%)	0 (0.00%)	0.12	F = 2.37
Chorioamnionitis	10 (3.69%)	3 (0.93%)	0.02	F = 5.19
Prolonged delivery	10 (3.69%)	0 (0.00%)	≤ 0.05	F = 14.10
Tachycardia	11 (4.05%)	1 (0.31%)	≤ 0.05	F = 0.37
Amniotic liquid	18 (6.64%)	4 (1.24%)	< 0.05	F = 12.02
Placenta	8 (2.95%)	0 (0.00%)	0.05	F = 9.60
Cord	3 (1.10%)	0 (0.00%)	0.05	F = 3.57

*Note:* The F statistic is associated with the chi‐square test, while the t statistic pertains to the independent sample t‐test.

Abbreviations: GBS, Group B streptococcus; UTI, urinary tract infections.

### 3.2 Neonatal Characteristics

Five hundred and ninety‐two neonates with a mean age of 3.99 ± 5.42 days took part in this study. Of this total, 45.77% (271) presented with early onset sepsis, with 45.78% (271) had a positive blood culture. (Table 1). There was a statistically significant differences between the groups in terms of head circumference (t = 2.57 *p*‐value = 0.01), body weight (t = 6.07 *p*‐value < 0.05) and height (t = 5.23 *p*‐value < 0.05). All measurements were higher in the group negative than in the group positive (Table 2). The proportion of boys in group positive were 59.04% (160) and, 52.95% (170) in the group negative. Among the neonates with EON sepsis, 22.14% (60) had a first minute Apgar score below 7, while10.28% (33) neonates without sepsis had Apgar score below this threshold. These differences were statistically significant (F = 12.72 *p*‐value = 0.02). Half, 49.81% (135) of the neonates with sepsis had a 5th minute Apgar score < 7, compared to 23.05% (74) of neonates without sepsis, which was statistically significant (F = 13.65 *p*‐value = 0.01). Symptoms like vomit, cold, lethargy, poor nutrition, and intubation were frequently more prevalent in group positive than in negative. In contrast, apnea, tachypnea and cyanosis were more frequent in the group negative.

**Table 2:** Neonatal socio demographic and clinical signs.

Variables	Group positive (*n* = 271)	Group negative (*n* = 321)	*p* value	Statistics
Sex (Boy: Girl)	160 (59.04%): 110 (40.59%)	170 (52.95%): 151(47.04%)	0.17	F = 3.54
Birthweight (g)	2014.37 ± 869.52	2460.21 ± 912.24	< 0.05	t = 6.07
Head circumstance (cm)	30.52 ± 4.59	33.27 ± 18.40	0.01	t = 2.57
Height (cm)	43.51 ± 7.21	46.41 ± 5.97	< 0.05	t = 5.23
First‐minute Apgar scores less than 7	60 (22.14%)	33 (10.28%)	0.02	F = 9.28
Fifth‐minute Apgar scores less than 7	135 (49.81%)	74 (23.05%)	0.01	F = 8.94
Perinatal care	228 (84.13%)	310 (96.57%)	< 0.05	F = 26.42
Tachycardia	10 (3.69%)	9 (2.80%)	0.54	F = 0.37
Anomalies	6 (2.21%)	16 (4.98%)	0.07	F = 3.15
Skin disorder	3 (1.10%)	9 (2.80%)	0.14	F = 2.13
Lethargy	41 (15.12%)	27 (8.41%)	0.01	F = 6.52
Low nutrition	28 (10.33%)	9 (2.80%)	< 0.05	F = 14.21
Vomit	19 (7.01%)	5 (1.55%)	< 0.05	F = 11.23
Apnea	44 (16.23%)	85 (26.47%)	< 0.05	F = 9.04
Tachypnea	32 (11.80%)	125 (38.94%)	< 0.05	F = 55.51
Cyanosis	87 (32.10%)	151 (47.04%)	< 0.05	F = 13.63
Hypothermia	76 (28.04%)	34 (10.59%)	0.04	F = 5.19
Have convulsion	51 (18.81%)	74 (23.05%)	0.21	F = 1.54
Have jaundice	33 (12.17%)	69 (21.49%)	0.05	F = 5.10
Intubation	105 (38.74%)	48 (14.95%)	< 0.05	F = 16.23
Death	84 (30.99%)	29 (9.03%)	0.03	F = 8.52

*Note:* The F statistic is associated with the chi‐square test, while the t statistic pertains to the independent sample t‐test.

### 3.3 Antibiotic Susceptibility Patterns of Isolated Bacterial Species

The predominant, 82.28% (229) of the bacteria group isolated from the neonates who presented with early onset sepsis were the Gram‐positives, of which 60.14% (163) were *S. aureus* and 24.35% (66) being *Coagulase‐negative staphylococci* (CoNS).

As presented in Table 3, species‐specific antibiotic resistance rates among bacterial isolates from neonates with sepsis revealed distinct patterns for both Gram‐positive and Gram‐negative pathogens. For Gram‐positive bacteria, *S. aureus* exhibited a high level of resistance to commonly used beta‐lactam antibiotics. 83(50.92%) of the *S. aureus* isolates tested were resistant to penicillin and 67 (41.10%) to ampicillin. Among 23 *E. coli* isolates identified, 14 (60.86%) demonstrated resistance to ampicillin, highlighting high resistance rate to this first‐line antibiotic. However, resistance to other antibiotics was notably lower; no isolates (0/23, 0.00%) were resistant to ciprofloxacin or amikacin, indicating full susceptibility to these agents. Conversely, resistance to Tetracycline was observed in 4 isolates (17.39%), reflecting a moderate resistance for this antibiotic.

**Table 3:** Antimicrobial resistance patterns of Gram positive and negative bacteria from blood cultures in patients with neonatal sepsis.

Antibiotics	*S. aureus* (*n* = 163)	CoNS (*n* = 66)	*K. pneumonia* (19)	*E. coli* (*n* = 23)
Clindamycin	13 (7.97%)	5 (7.75%)	‐‐‐	‐‐‐
Cefoxitin	44 (26.99%)	25 (38.87%)	‐‐‐	‐‐‐
Erythromycin	60 (36.80%)	28 (42.42%)	‐‐‐	‐‐‐
Oxacillin	44 (26.99%)	25 (38.87%)	‐‐‐	‐‐‐
Penicillin	83 (50.92%)	43 (65.15%)	‐‐‐	‐‐‐
Trimethoprim‐sulfamethoxazole	60 (36.80%)	30 (45.45%)	8 (42.10%)	7 (30.43%)
Tetracycline	54 (33.12%)	28 (42.42%)	‐‐‐	‐‐‐
Ampicillin	67 (41.10%)	25 (38.87%)	18 (94.73%)	14 (60.86%)
Ceftriaxone	40 (24.53%)	20 (30.30%)	15 (78.94%)	6 (26.08%)
Chloramphenicol	50 (30.67%)	25 (38.87%)	‐‐‐	‐‐‐
Gentamicin	60 (36.80%)	35 (53.03%)	3 (15.78%)	2 (8.69%)
Ciprofloxacin	32 (19.63%)	13 (19.69%)	3 (15.78%)	0 (0.00%)
Amoxicillin‐clavulanate	‐‐‐	‐‐‐	6 (31.57%)	6 (26.08%)
Tetracycline	‐‐‐	‐‐‐	7 (36.84%)	4 (17.39%)
Amikacin	‐‐‐	‐‐‐	2 (10.52%)	0 (0.00%)
Ceftazidamide	‐‐‐	‐‐‐	2 (10.52%)	2 (8.69%)
Chloramphenicol	‐‐‐	‐‐‐	6 (31.57%)	7 (33.43%)

Abbreviations: CoNS, coagulase‐negative staphylococci; *E. coli*, *Escherichia coli*; *K. pneumonia*, *Klebsiella pneumonia*; *S. aureus*, *Staphylococcus aureus*.

## 4 Discussion

This study aimed to identify the neonatal and maternal characteristics associated with neonatal sepsis, as well as to determine the microbial profile and antibiotic susceptibility patterns of bacteria isolated from septic cases, focusing on commonly prescribed antibiotics used in the two health facilities in Iran.

In the current study, less than half of the infants (45.77%) presented with early‐onset sepsis, a proportion lower than the 81.82% reported in a study conducted in Gondar, Ethiopia, the 81.41% reported in a study from Bishoftu, Ethiopia, and the 76.90% reported in another study from Ethiopia [24, 25, 26] were higher than the 15% early‐onset sepsis rate reported in a cohort from the Ahafo Region of Ghana [27]. These variations in EOS prevalence across studies may reflect differences in study populations, healthcare practices, or regional factors. For instance, the lower rate in the Ahafo Region of Ghana, could be linked to their setting in a developed country with advanced neonatal care and infection control measures, whereas the higher rates in Ethiopian studies may relate to limited access to prenatal care or higher rates of perinatal infections. However, further research is needed to determine the exact reasons for this variability.

In this study, neonates with positive blood cultures for neonatal sepsis exhibited symptoms such as fever, fatigue, vomiting, and malnutrition. These symptoms are consistent with early‐onset sepsis findings reported in other studies [28, 29, 30, 31]. The link between neonatal sepsis and symptoms like fever, fatigue, and vomiting likely reflects the pathophysiology of bacterial spread in the bloodstream. Fever and fatigue result from cytokine release and the immune system's metabolic demands, while vomiting may arise from gastrointestinal involvement or systemic toxicity [32]. Malnutrition may increase the previous susceptibility to neonatal sepsis by weakening host defenses or arise as a secondary effect of prolonged illness and reduced nutritional intake. Other study have similarly identified malnutrition as a common comorbidity in sepsis, heightening the risk of adverse outcomes [33].

The findings of the present study indicate that a history of maternal urinary tract infection (UTI) is associated with an increased risk of neonatal sepsis. This observation aligns with existing evidence suggesting a link between maternal infections and neonatal bacterial outcomes. For instance, Molla Fenta et al, (2022) reported that neonates born to mothers with a UTI during pregnancy had 19 times higher odds of developing a bacterial infection compared to those born to mothers without UTI [34]. This relationship may be attributed to multiple factors, including bacterial transmission from mother to neonate, alterations in maternal microbiota, and systemic inflammatory responses during pregnancy [35]. Also, Siakwa et al reported that the history of urinary tract infections is strongly associated with the likelihood of neonatal sepsis. Women with a history of urinary tract infections (UTIs) were almost four times more likely to have babies who developed neonatal sepsis than those without prior UTIs [28]. This association may be due to maternal infections leading to colonization of the birth canal or amniotic fluid, increasing the risk of vertical transmission of pathogens to the neonate. Screening and timely treatment of UTIs during pregnancy could help reduce the risk of neonatal sepsis.

Similar to findings in the presented study, Siakwa et al found that the neonate's apgar score at 1 min after birth was strongly associated with the likelihood of developing neonatal sepsis [28]. Neonates with low apgar scores may exhibit weakened immune responses or impaired systemic resilience, making them more susceptible to bacterial invasion—whether from gram‐positive pathogens, as seen in the findings of this study, or other causative agents [9]. This connection is particularly relevant in the context of early sepsis symptoms (e.g., fever, fatigue, vomiting, and malnutrition) observed in our positive study group, which could be exacerbated by the initial vulnerabilities reflected in a low apgar score.

This study demonstrated that neonatal sepsis was significantly associated with lower birth weight, with affected neonates weighing less than those without sepsis. Similarly, other studies have reported that infants weighing less than 2.5 kg were approximately a 6.82‐fold higher risk of developing neonatal sepsis compared to those weighing more than 2.5 kg [36, 37, 38].

Our results show that neonates exposed to amniotic fluid or umbilical cord infections were more likely to develop sepsis, which may be due to the vertical transmission of pathogens from the mother to the infant during pregnancy or delivery. This finding aligns with prior studies that have reported similar links between maternal or perinatal infections and adverse neonatal outcomes [39, 40, 41].

“In our investigation S. aureus and other gram‐positive pathogens were the predominant isolates, which is contrary to findings from other studies, which have primarily identified gram‐negative pathogens [31]. In other studies, indicated Klebsiella, *S. aureus*, and *E. coli* were associated with neonatal sepsis [3, 42]. The findings of this study revealed a higher prevalence of gram‐positive isolates compared to Gram‐negative ones, similar pattern also observed in studies from Nigeria and Kuwait [43, 44]. The predominance of gram‐positive bacteria could have several implications. It may reflect selective pressure from antibiotics commonly used in these regions, which are more effective against gram‐negative species, thereby enabling gram‐positive pathogens to gain a foothold. Alternatively, it could indicate differences in infection control practices or patient populations that favor the transmission or persistence of gram‐positive organisms. Understanding these underlying drivers are critical, as they could inform tailored therapeutic strategies and antibiotic stewardship programs to address this shift effectively. The divergence seen in current results and those from Nigeria and Kuwait highlight the importance of regional variability in microbial epidemiology and the need for localized surveillance to guide clinical decision‐making. Further comparative analyzes across diverse settings could help clarify whether this pattern is a widespread phenomenon or specific to certain ecological or healthcare contexts. A study demonstrated that gram‐positive bacteria, such as GBS, are more frequently associated with neonatal sepsis [45].

The findings of this study indicate that *S. aureus*, a gram‐positive bacterium, was prevalent in the study group and exhibited high sensitivity to Clindamycin, Ciprofloxacin, Ceftriaxone, Oxacillin, and Cefoxitin. These findings align with observations from other regions, where S. aureus and CoNS isolates commonly display resistance to penicillin and ampicillin, as well as multidrug resistance [46]. It is important to consider that the rate of resistance was lower in this study than in other studies by Eshetu and Clock [24, 25]. Moreover, Methicillin‐resistant Staphylococcus aureus (MRSA) and vancomycin‐resistant Enterococci (VRE) account for 4% and 1% of infants who discharge from NICU in the United States [47]. Excessive antibiotic use during pregnancy may drive antibiotic resistance, fostering the emergence of treatment‐resistant bacterial strains. This practice has been associated with the increasing prevalence of resistant Escherichia coli strains, which are frequently implicated in neonatal sepsis, especially in extremely preterm infants [3, 48, 49].

The higher proportion of drug resistance observed in this study, along with findings from other research, underscores a growing concern regarding the rapid rise in neonatal antibiotic resistance, which poses a significant threat to effective treatment outcomes. The findings necessitate careful interpretation particularly in distinguishing pollutants from CoNS infections. Accurate diagnosis remains challenging due to the lack of clear symptoms and standardized criteria. Furthermore, the potential for contamination in blood cultures can lead to prolonged hospitalization and unwarranted antibiotic use. Although intensive research has reduced neonatal mortality and morbidity, bacterial isolation underscores the urgent need for local infection and resistance pattern monitoring. The effective implementation of antibiotic stewardship programs is crucial. Finally, updating treatment recommendations is essential, considering facility conditions, infection prevalence, and the presence of multidrug‐resistant pathogens [50].

This study has several limitations. The use of aerobic culture methods alone may have missed anaerobic pathogens involved in bloodstream infections, and also focusing solely on blood samples may have limited the detection of other bacterial pathogens.

## 5 Conclusion

This study identified key risk factors for neonatal sepsis included low birth weight, inadequate prenatal care, and symptoms such as tachypnea and cyanosis. Also, it was found Staphylococcus aureus and CoNS as the primary causes of early‐onset neonatal sepsis. Antibiotic susceptibility testing showed that over 91% of *S. aureus* isolates were resistant to penicillin and ampicillin. To mitigate infection risk and curb antibiotic resistance, we recommend integrating robust infection control measures into neonatal care and enhancing antenatal screening protocols.

## Author Contributions

All authors have read and approved the final version of the article. The corresponding author, Amir Emami, had full access to all data in the study and assumes full responsibility for the integrity and accuracy of the data analysis. Amir Emami designed the study and drafted the initial article. Fatemeh Javanmardi conducted the statistical analysis, edited the article, and performed the revisions. Zahra Hashemi provided scientific comments, while A.E. carried out the laboratory tests. Atila Erami and Zeynab Noeizad contributed to revising the article.

## Ethics Statement

IR.SUMS.REC.1400.830.

## Conflicts of Interest

The authors declare no conflicts of interest.

## Transparency Statement

The lead author Amir Emami affirms that this manuscript is an honest, accurate, and transparent account of the study being reported; that no important aspects of the study have been omitted; and that any discrepancies from the study as planned (and, if relevant, registered) have been explained.

## Data Availability

Data are available upon reasonable request from the corresponding author.
